# Eyelid laxity and sleep apnea syndrome: a review

**Published:** 2019

**Authors:** Razvan Cristescu Teodor, Florin Dumitru Mihaltan

**Affiliations:** * Centre Hospitalier Bourges, France; ** ”Marius Nasta Pneumophtisiology Institute, Bucharest, Romania; “Carol Davila” University of Medicine and Pharmacy, Bucharest, Romania

**Keywords:** sleep apnea syndrome, floppy eyelid syndrome

## Abstract

The purpose of this paper is to review the association between a medical entity called the floppy eyelid syndrome (FES) and a very serious respiratory disease with repercussions on various other body organs and systems: the obstructive sleep apnea syndrome. The epidemiology, pathophysiology, and treatment of these two diseases are intertwined but still not enough recognized. Eye disorders affect a great number of patients in modern societies and the cause of their suffering is often left undiscovered, practitioners preferring a symptomatic treatment. However, the ophthalmologist should be aware of the possibility of a sleep disorder in their patients with certain pathologies, as well as sleep physicians, who should be aware of the possibility of eye problems and refer them for a checkup. Finally, a review of literature is undergone, evaluating the possibility that the treatment of one or the other disease may benefit both.

## Introduction

Obstructive sleep apnea syndrome (OSAS) is an underdiagnosed disease with important systemic implications. It is characterized by interruption in ventilation for more than 10 seconds, because of the collapse or narrowing of the superior respiratory airways [**1**].

It is estimated that sleep apnea syndrome, defined as apnea/ hypopnea index greater or equal with 5 has a prevalence of 20% in males and 10% in females [**2**]. Consequently, to years of sleep fragmentation and deprivation, almost every bodily organ can be affected, including the eye, and patients often present to non-sleep specialists complaining of symptoms caused or exacerbated by OSAS [**3**]. The recognition of these manifestations has passed, in the last years, from obscurity into regular clinical practice [**4**].

The main risk factors for developing OSAS are the following: male gender, obesity, upper airways anomalies, alcohol consumption, snoring, sedative use, great neck width/ circumference [**5**]. Among others, the main symptoms of the disease are excessive daytime sleepiness, focus/ concentration difficulties, memory problems, and morning and daytime headaches.

Sleep apnea is associated with pulmonary hypertension, myocardial infarction, cardiac arrhythmia, congestive heart failure, stroke, death of cardiac causes, and death of any causes. Obstructive sleep apnea syndrome was identified as a secondary treatable cause of arterial hypertension by the Reunited National Committee for prevention, detection, evaluation, and treatment of hypertension [**6**].

Obstructive sleep apnea syndrome influences many aspects of physiological functions, affecting pulmonary, cardiovascular, and cerebrovascular systems. Multiple associations have been made between sleep apnea and some ocular diseases as diverse as glaucoma, non-arteritic ischemic optic neuropathy, bilateral disc oedema, floppy eyelid syndrome, blepharitis, ptosis, papillary conjunctivitis, filamentary keratitis, vascular retinal tortuosity, central serous chorioretinopathy.

Although authors have speculated about many hypotheses, it is not known to this day how exactly each of the conditions listed above correlates with the obstructive sleep apnea syndrome.

The floppy eyelid syndrome (FES) is a palpebral laxity disorder initially described by Culbertson and Ostler in 1981 and characterized by the presence of a lax superior lid associated with papillary tarsal conjunctivitis in young men with obesity [**7**]. Many other studies have described this phenomenon since then, including the ones made by Van den Bosch [**8**] and Fowler [**9**].

The floppy eyelid syndrome is characterized by the presence of easily evertible eyelids in the context of a papillary conjunctivitis. According to some studies, up to 90-100% of the patients with this syndrome also have obstructive sleep apnea syndrome [**10**-**12**]. It remains an under-diagnosed disorder with unclear pathogenesis.

## Epidemiology of FES and OSAS association

Gonnering and Sonneland made the oldest association between the obstructive sleep apnea syndrome and the “floppy eyelid syndrome” in 1987 [**13**]. The result from a meta-analysis in 2017 suggested that OSAS patients have 4.12 times higher FES risk compared to those non-OSAS individuals [**14**].

In 1997, McNab reported a series of 50 patients with floppy eyelid syndrome, 48 of whom (96%) had a history of sleep breathing disorders. 27 patients underwent polysomnography, and in 26 of these (96%), the presence of sleep apnea syndrome was confirmed [**10**].

Increased palpebral laxity was associated with obstructive sleep apnea syndrome and, in addition, it was shown to have a positive correlation with disease severity [**12**,**15**,**16**].

In one study, floppy eyelid syndrome was frequently associated with OSAS compared with healthy individuals, but there was no correlation found between obesity and FES. Nevertheless, the presence of lid hyperelasticity in OSAS patients with high BMI was statistically significantly increased when compared with low-BMI OSAS patients [**17**].

One study by Mojon, Goldblum et al. researched, among other things, to determine if ocular irritation symptoms are more prevalent in patients with high apnea/hipopnea index (AHI), but these showed to be rare. The tear break-up time (TBUT), a measurement of dry eye, established by instilling a drop of fluorescein on the eye, examining at the slit lamp and determining the time necessary for patches devoid of fluorescein to appear, demonstrated a negative correlation with the AHI. In addition, patients with more severe sleep apnea had a higher prevalence of floppy eyelid syndrome. Also, they found a correlation with a bigger superior eyelid distraction distance and lacrimal gland prolapse. In contrast, corneal surface anomalies were not found. Examinations rarely revealed cases like bilateral keratoconus and bilateral Fuchs dystrophy [**12**].

Krager, White et al. tried to determine the prevalence of the “floppy eyelid syndrome” in patients with OSAS, examining 59 patients by performing polysomnography, after which 44 patients were discovered with OSAS and 15 without. The number of apnea/ hypopnea episodes per hour was recorded. The presence of “floppy eyelid syndrome” was defined by subjectively easy eversion of the upper eyelid, tarsal papillary conjunctivitis on slit lamp examination and lash ptosis. It was quantified by measuring the necessary force to displace the upper lid away from the globe by 5 mm with a specially designed instrument. Among examined patients, there was a newly case of “floppy eyelid syndrome”, and, as another patient, was already diagnosed before entering the study, the total prevalence rose to 4,5% (2 out of 44). Subjectively easily everted superior eyelid was much more common in patients who tested positive for the respiratory disease. Adjusted for age and BMI, there was a trend for the association between OSAS and easily eversion of the eyelid, but without statistical significance. The force necessary to displace the upper eyelid with 5 mm was smaller in patients with easily evertible eyelids, but it was not associated with sleep apnea syndrome or with the apnea/ hypopnea index. The conclusions of this study were that “the prevalence of the floppy eyelid syndrome in OSAS is low, but the prevalence of isolated palpebral laxity is high. Patients with easily evertible upper eyelids are at risk of developing sleep apnea syndrome” [**15**].

In 2012, Chambe J, Laib S et al. tried to determine if the severity of obstructive sleep apnea syndrome is associated with floppy eyelid syndrome in a prospective study of 127 patients aged 27 to 75. Variables like age, BMI, and proportion of male patients increased with disease severity. The presence of floppy eyelid syndrome was confirmed in 15,8% of the patients without sleep apnea syndrome and in 25,5% of all the patients diagnosed with OSAS. The proportion was even higher when only severe forms of OSAS (AHI>=30/ h) were considered. A significant correlation between OSAS severity and floppy eyelid syndrome was found after adjusting for age, sex, and BMI. The results suggested that “severe OSAS is an independent risk factor for the floppy eyelid syndrome”. These two diseases might have common biological determinants like tissue elasticity. The recognition of such an association by clinicians is important in order to correctly diagnose and treat patients [**18**].

Muniesa et al. tried to determine the correlation bidirectionally. They took on one-side patients with sleep apnea for which they determined the prevalence of palpebral hyperlaxity and on the other side patients already diagnosed with FES, for which they practiced some polysomnography studies. 

A significantly higher incidence of palpebral hyperlaxity had been found in patients with the sleep disorder than in the ones without this pathology. In the second part, 38 out of 45 patients (85%) with floppy eyelid syndrome were diagnosed with OSAS, and 65% had the severe form (AHI>30). The conclusions were that “OSAS might be an independent risk factor for eyelid hyperlaxity and severe OSAS is common in patients with FES” [**19**].

Acar et al. investigated the link between ocular surface problems and the severity of the disease in patients with OSAS (**Table 1**). Sleep apnea syndrome, particularly the moderate and severe forms, was associated with low values of the Schirmer test and TBUT (tear break-up time), and with high values of the OSDI questionnaire (a questionnaire that grades the severity of ocular surface symptoms) and higher corneal staining pattern stage. A positive correlation between obstructive sleep apnea syndrome and LES (laxity eyelid syndrome) was also revealed [**20**,**21**].

**Table 1:** Modified after [**21**] - Loyola Chicago University

Clinical finding	Control group, non-OSAS	OSAS mild	OSAS moderate	OSAS severe	Statistical significance (p<0,5)
FES	23,1%	41,7%	66,7%	74,6%	p<0,01
Schirmer Test (mm)	10,76 +/ - 3,58	9,83 +/ - 2,53	7,73 +/ - 2,42	6,97 +/ - 2,15	p <0,01
TBUT (sec)	10,53 +/ - 3,64	9,46 +/ - 2,40	7,29 +/ - 2,13	6,82 +/ - 2,20	p <0,01
OSDI	12,57	22,90	45,94	56,68	p<0,01
Corneal stain	0,26	0,40	0,98	1,14	p<0,01

## Pathophysiology of Floppy eyelid syndrome

Lid and ocular surface disturbances are considered secondary to the mechanical effects of eye rubbing and facedown posture during sleep, which determines palpebral and conjunctival contact with the pillow.

Direct trauma induces chronic inflammation and tissular ischemia with a possible supra-expression of elastolytic enzymes, the most well known of which being the matrix metalloproteinases [**22**].

Histologic studies have shown a loss of elastin fibers with supraexpression of elastolytic proteases in the tarsal plates of the eyelids, feature presumed to be mediated by mechanical stress and/ or alternating ischemia/ reperfusion lesions [**23**,**24**]. Hypoxia and later reperfusion can lead to the supraexpression of matrix metalloproteinases [**25**].

Netland et al. have shown a reduction of tarsal elastin in the lid structure of patients with floppy eyelid syndrome, feature that sustains the hypothesis mentioned above [**23**]. He analyzed eight patients with floppy eyelid syndrome, four of whom undertook lid-shortening surgery. Microscope examination of lid fragments taken after surgery revealed chronic conjunctival inflammation, papillary conjunctivitis, and Meibomian gland anomalies (granuloma formation). An interesting fact observed was the marked decrease of elastin fiber quantity in tarsal plates of patients with floppy eyelid syndrome, compared with the control group. On the other hand, the quantity and quality of tarsal collagen fibers was comparable between groups [**23**].

Another fact worth mentioning is the presence of asymmetric disease, corresponding to the patient’s position during sleep, as well as the disappearance of symptoms after using eye shields [**10**].

Schlotzer-Schrehardt U, Stojkovic M et al. also investigated histologically the lids of patients with floppy eyelid syndrome [**24**]. Their study demonstrated a loss of elastin fibers and the overexpression of elastin-degrading enzymes in the tarsal plates. These modifications are said to be caused firstly by a mechanical factor and secondly by an alternance of ischemia and reperfusion of the respective tissues. The objective was to histopathologically investigate and to analyze the potential alteration of palpebral biopsy specimens in patients with FES, paying special attention to the content of elastic fibers, their ultrastructure, and the expression of elastin degrading amines. Specimens obtained from lower lid surgery was examined at microscope and immunohistochemically and revealed a decrease of elastin in the tarsus and lid skin of patients with floppy eyelid syndrome. It was shown that residual elastic fibers were abnormal ultrastructurally. Additionally, immunohistology found high matrix metalloproteinase levels in areas with elastic depletion in patients with floppy eyelid syndrome, in comparison with the control group.

It was concluded that the “upregulation of proteins and elastolytic enzymes leads to elastic fiber degradation and subsequently to tarsal laxity and lash ptosis” [**24**].

MMP-9 is an important inflammatory marker also found in the lacrimal film of the patients with dry eye. In one study, 89% of the patients with palpebral laxity had a positive tear film essay for this enzyme. This strong, statistically significant association additionally reinforces the role of MMP-9 in disease pathology [**21**].

## Pathophysiology of OSAS

The pathophysiology might vary considerably between patients and is not fully understood, but some general mechanisms can be found.

The upper respiratory airway contains a large number of muscles and soft tissues but lacks rigid or bony support. It presents an easily collapsible segment between the hard palate and the larynx [**26**].

An important mechanism in OSAS pathology is the interaction between the pharyngeal anatomy and a diminished ability of dilator muscles to maintain the airways opened [**27**].

Narrow superior airways predispose to collapse. Measured with CT or RMI, their diameter is reduced in OSAS patients compared to normal individuals [**28**-**30**]. The surrounding soft tissue is altered in patients suffering from OSAS, putting them at risk for airway collapse [**28**]. In addition, during a study performed by Isono et al., it was noticed that during general anesthesia, superior airways are more easily collapsible in OSAS affected individuals [**31**].

During wakefulness, however, these patients compensate by way of some protective mechanisms: reflexes that increase the activity of the upper airway dilator muscle [**27**]. Accordingly, during this period, the genioglossus, the biggest and most studied airway dilator muscle in humans, has a more intense activity in OSAS affected subjects compared to normals [**26**]. On the other hand, the muscular tone measured with intramuscular EMG is reduced in OSAS patients during sleep and in normal individuals only at the beginning of sleep [**32**,**33**]. In comparison with the sleep transition period, slow wave sleep is associated with an increased activity of the airway dilator muscles. Thus, when patients are able to achieve slow wave sleep, increased upper airway dilator muscle activity may be one important factor contributing to the improvement in apnea severity that is commonly observed in this sleep stage [**34**].

The geometry and the caliber of the airways differ in patients with OSAS compared to normals. The apneic airway is smaller and narrower in the lateral diameter. No anteroposterior narrowing was discovered on examination. The difference was not explained by the bony structure but by the bigger pharyngeal walls in the OSAS patients. The major anatomical factor was the thickening of the muscular wall and not an enlargement of the parapharyngeal adipose tissue [**35**].

It is known that CPAP (continuous positive air pressure) treatment enlarges the diameter of the superior airways. Progressively increasing the CPAP pressure produces a bigger volume and area in the retropalatine and retroglossal regions. Enlargements in the lateral dimension were bigger than in the anteroposterior one. The thickness of the lateral pharyngeal wall decreased and the distance between the lateral adipose pads increased. These data suggest that lateral pharyngeal walls are more compliant than the soft palate and tongue [**36**].

It can be assumed that mechanisms involved in determining palpebral laxity are also concerned in pharyngeal lateral wall laxity.

A study compared the components of the extracellular matrix of the lateral muscular pharyngeal wall in normal patients and in OSAS affected ones. Samples obtained after pharyngeal surgery were analyzed histochemical and immunologically. They revealed an increase in collagen type 1 according to age and presence of OSAS. MMP-1 had a variable expression between individuals but did not differ significantly between groups. A quantitative deterioration of elastic fibers was not detected, but an ultrastructural level alteration cannot be excluded. The absence of inflammatory signs on the harvested samples suggests that the inflammatory processes might be localized in the tissues that line the airways and not in the deeper ones [**37**].

Another study described the presence of inflammatory cells in great amounts in mucosal and muscular pharyngeal tissues in OSAS patients compared with normals. Immunohistological studies for neuronal or muscular membrane markers revealed superior airway muscle denervation in OSAS individuals [**38**].

Sullivan et al. described an increased prevalence of OSAS in patients with Marfan syndrome, a genetic disease that causes muscular flaccidity and increased collapsibility of pharyngeal walls due to elastic fiber anomalies [**39**].

## Evolution

Obstructive sleep apnea is a potentially fatal disease. The apneic and hypopneic episodes can cause systemic and pulmonary hypertension, leading to congestive cardiomyopathy and cardiac arrhythmia risk.

Concerning the eye problems, nocturnal eyelid eversion can lead to chronic conjunctivitis, corneal erosions, ulceration, neovascularization, and scarring, and in the most advanced cases permanent decreased vision. A combined medical and surgical approach in often successful in managing floppy eyelid syndrome. 

## Treatment

Lubricating or antibiotic ointments can be enough for mild corneal and conjunctival abnormalities determined by the lax and easily evertible floppy eyelids. Taping the eyelids at nighttime or wearing an eye shield during sleep is useful. Nevertheless, conservative medical treatment is often inadequate in relieving symptoms. Frequently, surgical intervention is required: lateral tarsal strip procedures [**40**], full-thickness horizontal shortening of the lateral 1/ 4-1/ 3 lid margin [**41**,**42**], and pentagonal full-thickness resection [**43**].

Continuous positive air pressure therapy (CPAP) is the standard treatment option for OSAS and can generally reverse the condition if appropriate titration is used. Although treatments for OSAS and FES, taken separately, are well known and straightforward, the hypothesis that CPAP could improve FES or other ocular symptoms was rarely investigated.

In 2000, McNab AA described a case of reversal of floppy eyelid syndrome in a patient with obstructive sleep apnea syndrome who was treated by CPAP. The respective 32-year-old male patient with only left-side FES underwent treatment with positive air pressure by mask for 4 years after which ocular signs and symptoms disappeared [**44**].

Acar M et al. assessed the long-term effects of 18 months of positive airway pressure (PAP) therapy on the eyes. The pre- and post-treatment values for eye examination scores (presence of floppy eyelid syndrome, results of the Ocular Surface Disease Index - OSDI - questionnaire, Schirmer I test, tear film break-up time values, and corneal staining stages) were recorded and compared. The study investigated 17 patients with moderate OSAS and 34 with severe OSAS. After the 18 months of treatment, FES stage was lower, the TBUT and Schirmer test values were higher, OSDI was lower after treatment, and so was the corneal staining stage. In conclusion, “long-term PAP therapy (at least one year) might improve the clinical picture of FES probably by providing a return to normal sleep patterns” [**45**].

It was shown that even if CPAP users had similar upper and lower lid laxity as non-CPAP users, the first ones had a better tear film and less ocular irritation. The more stable tear film observed in CPAP treated patients was probably secondary to supine sleeping position, necessary during this type of treatment [**4**].

CPAP treatment had also reported negative effects on eyes, as in some cases, ocular irritation due to air leaks from the mask edges was noticed [**46**]. In one study, 20% of the users experienced conjunctivitis secondary to air leaks from the mask edges [**4**].
